# National implementation of vaginal Natural Orifice Transluminal Endoscopic Surgery for benign hysterectomies: A historical cohort study of Swedish data 2021–2023

**DOI:** 10.1111/aogs.70142

**Published:** 2026-01-14

**Authors:** Johanna Wagenius, Sophia Ehrström, Karin Källén, Jan Baekelandt, Andrea Stuart

**Affiliations:** ^1^ Department of Obstetrics and Gynecology, Institute of Clinical Sciences Lund University Lund Sweden; ^2^ Department of Obstetrics and Gynecology Helsingborg Hospital Helsingborg Sweden; ^3^ Division of Obstetrics and Gynecology, Department of Clinical Sciences Karolinska Institutet, Danderyd Hospital Danderyd Sweden; ^4^ Considra Gyn Nacka Hospital Nacka Sweden; ^5^ Department of Clinical Sciences, Center of Reproduction, Epidemiology, Tornblad Institute Lund University Lund Sweden; ^6^ Department of Obstetrics and Gynecology Imelda Hospital Bonheiden Belgium; ^7^ Department of Obstetrics and Gynecology, Development and Regeneration University of Leuven Leuven Belgium

**Keywords:** complications, minimally invasive surgery, surgical outcome, vNOTES hysterectomy

## Abstract

**Introduction:**

Vaginal Natural Orifice Transluminal Endoscopic Surgery (vNOTES) is a technique combining vaginal entrance to the abdomen with endoscopic overview. Previous studies have shown that vNOTES decreases operating time, hospitalization, postoperative complications, and pain. We aimed to present patient demographics, costs, and surgical outcomes following the implementation of vNOTES for benign hysterectomies in Sweden.

**Material and Methods:**

We conducted a historical cohort study with the first vNOTES hysterectomies in Sweden 2021–2023 involving 8 hospitals. Data was extracted from the Swedish National Quality Register for Gynecological Surgery (GynOp). Our main outcomes were intraoperative and postoperative complications, costs, and patient satisfaction. As a secondary objective, the odds ratios (OR) for any intraoperative or postoperative complication, respectively, were computed for BMI ≥30 versus <30, and for uterus weight ≥500 g versus <500 g.

**Results:**

A total of 545 patients were included in the study. The mean age of the patients was 49.9 ± 10.7 years. Of the included patients, 8.1% (*n* = 44) were nullipara, 19.3% (*n* = 105) had a BMI ≥30, 17.4% (*n* = 95) had a previous cesarean section, and 16.7% (*n* = 91) had other previous abdominal surgery. The median uterus weight was 148 g (interquartile range, IQR 86–299). The median surgical time was 65 minutes (IQR 48–91), and the median blood loss was 40 mL (IQR 25–90). Conversions to laparotomy occurred in 2% (*n* = 11), and reoperations occurred in 0.6% (*n* = 3) of the cases. The total intraoperative complication rate was 2.2% and the total postoperative complication rate was 8.4%. No significant differences in intraoperative and postoperative complications were found between BMI ≥30 and <30 and between uterus weight ≥500 and <500 g. Most of the patients (57.1%, *n* = 311) left the hospital the same day as the surgery. The 1‐year follow‐up after surgery showed that 90% of the patients were satisfied or very satisfied with the result.

**Conclusions:**

The implementation of vNOTES hysterectomies in Sweden has been safe showing similar complication rates compared to studies of other minimally invasive hysterectomy techniques. Surgical time, intraoperative bleeding, and conversions were in analogy with previous observational vNOTES studies. The 1‐year follow‐up after surgery showed high patient satisfaction.

## Graphical abstract

The implementation of vNOTES hysterectomy in Sweden shows low intra‐ and postoperative complication rates. The 1‐year follow‐up after surgery shows that 90% of the patients were satisfied or very satisfied with the result. vNOTES seems to be a safe method to introduce broadly in a national setting.

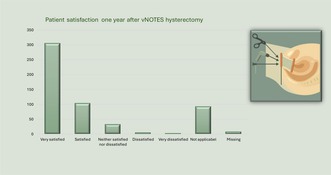

**Abbreviations**
AH: abdominal hysterectomy
BMI: body mass index
IQR: interquartile range
RH: robot assisted hysterectomy
TLH: total laparoscopic hysterectomy
VH: vaginal hysterectomy
vNOTES: vaginal Natural Orifice Transluminal Endoscopic Surgery

**Key message**

Following the implementation of vNOTES hysterectomy in Sweden, we found low risk of intra‐ and postoperative complications. vNOTES seems to be a safe method to introduce broadly in a national setting.

## 1 INTRODUCTION

In Sweden almost 4000 hysterectomies are performed annually on benign indications.^1^ There are several hysterectomy techniques which can be divided into three groups: abdominal, vaginal, and laparoscopic/robot assisted hysterectomy. A vaginal hysterectomy (VH) is preferable if possible since the patient recovers quickly, and it leaves no scar on the abdomen.^2^, ^3^, ^4^, ^5^ VH also has lower costs compared to other hysterectomy techniques.^6^, ^7^, ^8^ In contrast to surgical guidelines the incidence of vaginal hysterectomies has declined during the last decades, and hysterectomies are primarily performed robotically or laparoscopically.^8^, ^9^, ^10^ In 2021, 34.5% of all benign hysterectomies in Sweden were performed abdominally, 27.6% laparoscopically, 26.9% robotically, and 11.0% as a vaginal hysterectomy.^11^

Vaginal Natural Orifice Transluminal Endoscopic Surgery (vNOTES) is a technique that combines vaginal entrance to the abdomen with an endoscopic overview.^12^, ^13^, ^14^ The technique enables vaginal hysterectomy in patients with large uteri who previously were subject to abdominal hysterectomy.^15^, ^16^, ^17^ A vNOTES hysterectomy is performed by opening the vesico‐uterine pouch and the pouch of Douglas with the same technique as a vaginal hysterectomy. An Alexis retractor is inserted through the vagina and intraabdominally, and a gelport is attached to it. Pneumoperitoneum is created. Endoscopic instruments and a camera are inserted through the gelport which allows the surgeon to operate inside the abdomen through the vagina. If intraabdominal adhesions are present, the adhesiolysis can be performed during the endoscopic phase of the surgery. vNOTES also facilitates concomitant adnexal surgery. vNOTES is widely used for a great variety of benign indications for hysterectomy. The contraindications involve obstructed access to the pouch of Douglas in patients with previous rectal surgery, rectovaginal endometriosis, previous tubo‐ovarian abscess, and pelvic radiotherapy.

Previous studies have shown that vNOTES hysterectomy decreases operating time, hospitalization, postoperative complications, and pain compared to total laparoscopic hysterectomy.^18^, ^19^ Studies comparing vNOTES hysterectomy and vaginal hysterectomy show that vNOTES is an adequate alternative to VH, especially in nulliparous women or those with large uteri.^20^, ^21^

In Scandinavia, vNOTES was first implemented 2021 at the hospitals of Helsingborg/Ängelholm. Between 2021 and 2023, eight hospitals introduced the vNOTES technique in Sweden. In 2023 approximately 9% of hysterectomies were performed via vNOTES, which has become a more common technique than VH.^1^

The primary objective of this study was to describe patient demographics, costs, and surgical outcomes of the first vNOTES hysterectomies in Sweden. As a secondary objective we investigated if there was an association between intraoperative or postoperative complications and BMI or uterus weight.

## 2 MATERIAL AND METHODS

### 2.1 Data sources and study population

A historical cohort study was performed including the first vNOTES hysterectomies 2021–2023. Data was extracted from the Swedish National Quality Registry for Gynecological Surgery (GynOp). Inclusion criteria were all vNOTES hysterectomies with benign indication performed in Sweden from the implementation in September 2021 to December 2023. There were no exclusions. No control patients were registered in the registry.

The GynOp registry contains preoperative, intraoperative, and postoperative information regarding gynecological surgery since 1997. Data is collected from both the surgeon and the patient. Data entering is compulsory for surgeons, and the dataset is a complete set for the country with 90%–93% coverage during the period studied. All vNOTES surgeons (*n* = 23) are gynecologists specialized in gynecological surgery and have participated in a standardized vNOTES course before implementing the technique at their hospital.

The patient fills in a preoperative questionnaire to collect baseline demographic data and also fills in a postoperative questionnaire 8 weeks and 12 months after surgery regarding patient‐reported outcomes.^22^

vNOTES hysterectomies registered in the GynOp registry can also be registered in the iNOTESs case registry and the Swedish patient registry. Studies from these registries may include vNOTES cases.

### 2.2 Clinical and sociodemographic characteristics

Demographic and clinical variables were filled in at the time of the surgery (year of operation, age, body mass index (BMI), tobacco use, ASA classification, parity, previous abdominal surgery, previous cesarean section), type of surgery (hysterectomy and/or adnexal surgery), primary incision (abdominal, laparoscopic, vaginal), conversion to laparoscopy/laparotomy and indication for surgery. Directly postoperatively the surgeon registered data regarding uterus weight, blood loss, surgical time, complicating factors during surgery, conversions, and perioperative complications. Complicating factors during surgery were up to the surgeon's discretion to decide. It was filled in the GynOp registry with pre‐set tick‐box alternatives that include adhesions, obesity, large uterus/myomas, and endometriosis. The intraoperative complications were similarly classified by the surgeon as mild or severe by pre‐set tick‐box alternatives in the GynOp registry. The categorization of intraoperative complications in the GynOp registry is not validated so there can be misclassifications of degree of complication. After 8 weeks the surgeon confirmed or rejected the patient‐reported complications (including reoperations) in the registry according to the Clavien Dindo classification.^23^ Clavien Dindo I‐II were considered as mild postoperative complications and Clavien Dindo III‐IV as severe postoperative complications.

### 2.3 Costs

The intraoperative costs for the vNOTES set and sealing device were calculated. Costs related to surgical time and investment costs of the laparoscopic tower were not included. Data on costs were extracted from the financial department at Helsingborg Hospital, Sweden from 2022.

### 2.4 Statistical analysis

Descriptive statistics were presented as frequencies (percentage), or as mean (standard deviation, SD) and median (interquartile range, IQR) when appropriate. The odds ratios (OR) for any intraoperative or postoperative complication, respectively, were computed for BMI ≥30 as compared to BMI <30, and for uterus weight ≥500 g as compared to <500 g. ORs and 95% confidence intervals (CI) were retrieved using logistic regression. Due to small numbers only crude ORs were computed. All analyses were performed with the statistical software package IBM SPSS version 29.

Not applicable (N/A) answers were removed from the analysis of patient satisfaction. Missing data were presented but were excluded in the analysis mentioned above.

*p*‐values below 0.05 (two‐sided) were considered statistically significant.

## 3 RESULTS

In the database, 545 vNOTES hysterectomies were identified. Table 1 shows background characteristics. The mean age of the patients was 49.9 ± 10.7 years. Of the included patients, 8.1% (*n* = 44) were nullipara, 19.3% (*n* = 105) had a BMI ≥30, 17.4% (*n* = 95) had a previous cesarean section, and 16.7% (*n* = 91) had other previous abdominal surgery. The median uterus weight was 148 g (IQR 86–299). The smallest uterus weighed 26 g and the largest weighed 2935 g.

**TABLE 1:** Background characteristics of women undergoing vNOTES hysterectomy in Sweden 2021–2023.

	Cohort (*n* = 545)	
	*n*	%
Year of operation		
2021	21	3.9
2022	131	24
2023	393	72.1
Age		
Mean 49.9 ± 10.7 years		
≤30	6	1.1
31–49	292	53.6
50–69	219	40.2
≥70	28	5.1
Parity		
Nullipara	44	8.1
Vaginal delivery	381	69.9
Cesarean section	95	17.4
Missing	25	4.6
Previous abdominal surgery		
Yes	91	16.7
No	406	74.5
Missing	48	8.8
BMI		
<30	403	73.9
≥30	105	19.3
Missing	37	6.8
ASA class		
1	268	49.3
2	255	46.9
3	17	3.1
Missing	4	0.2
Smoking		
Yes	57	10.5
No	454	83.3
Missing	34	6.2

A total of, 64 patients (12%) had a uterus weight of over 500 g. The most common indications for surgery were dysfunctional menstrual bleeding (46.0%), cervical dysplasia (21.6%), and symptoms of pelvic pressure (12.8%).

During 2021–2023 23 surgeons at eight hospitals performed vNOTES hysterectomies in Sweden. The hospitals of Ängelholm/Helsingborg were the first to implement vNOTES and performed most of the cases, *n* = 347 (63.7%), followed by Considra clinic, *n* = 104 (19.1%). The other six hospitals performed the remaining 94 cases.

Table 2 shows surgical outcome and complications. The median surgical time was 65 min (IQR 48–91), and the median blood loss was 40 mL (IQR 25–90). Complicating factors during surgery were described in 6.2% of the cases, most commonly adhesions (*n* = 15), large uterus (*n* = 7), and obesity (*n* = 7). Conversions to laparotomy occurred in 2% (*n* = 11), and reoperations occurred in 0.6% (*n* = 3). Most of the patients (57.1%, *n* = 311) left the hospital the same day as the surgery.

**TABLE 2:** Surgical outcome and complications.

	Cohort (*n* = 545)	
	*n*	%
Duration of surgery		
Median 65 min (IQR 48–91)		
≤30	20	3.7
31–60	215	39.4
61–90	171	31.4
91–120	78	14.3
≥121	61	11.2
Uterus weight		
Median 148 g (IQR 86–299)		
≤300	406	74.5
301–500	67	12.3
501–1000	47	8.6
≥1001	17	3.1
Missing	8	1.5
Concomitant adnexal surgery	397	72.8
Bleeding		
Median 40 mL (IQR 25–90)		
0–500	530	97.2
501–1000	12	2.2
≥1001	3	0.6
Complicating factors during surgery	34	6.2
Complications—intraoperative		
Mild	7	1.3
Severe	5	0.9
Complications—postoperative		
Mild	36	6.6
Severe	10	1.8
Reoperations	3	0.6
Conversions to laparotomy	11	2.0
Days of hospitalization		
0	311	57.1
1	213	39.1
≥2	10	1.9
Missing	11	2.0

*Note*: Intraoperative complications: The surgeon defines the complications as mild or severe. Postoperative complications: Clavien Dindo I‐II are considered as mild postoperative complications and Clavien Dindo III‐IV as severe postoperative complications.

Abbreviations: IQR, interquartile range.

Mild intraoperative complications occurred in 1.3% (*n* = 7) and severe intraoperative complications in 0.9% (*n* = 5) of the cases. The most common mild intraoperative complication was bladder injury (*n* = 4) followed by intestinal injury (*n* = 2) and ureter injury (*n* = 1). Bladder injury was also the most common severe intraoperative complication (*n* = 3) followed by ureter injury (*n* = 2).

A mild postoperative complication was reported in 36 patients (6.6%). Urinary tract infection was the most common mild postoperative complication (*n* = 13), seven had a vaginal vault hematoma and five had unspecified fever. Other less common mild complications were wound infection, vaginal infection, and unspecified pain. A severe postoperative complication was reported in 10 patients (1.8%), most commonly vaginal vault hematoma/infection (*n* = 3), postoperative bleeding (*n* = 2), and fever/sepsis (*n* = 2). There were no reported deaths.

The number of intraoperative and postoperative complications were compared between BMI <30 and ≥30 and between uterus weight <500 and ≥500 g (shown in Table 3). No significant differences in intraoperative and postoperative complications were found between the groups. There was however a correlation between intraoperative blood loss and size of uterus.

**TABLE 3:** Odds of intra‐ and postoperative complications in women undergoing vNOTES hysterectomy (*n* = 545) by BMI and uterus weight.

	*N*	Intraoperative complications		Odds ratio for intraoperative complications	
		*n*	(%)	OR	95% CI
Total	545	12	(2.2)		
BMI (kg/m^2^)					
<30	403	9	(2.2)	1.0	Reference
≥30	105	2	(1.9)	0.85	0.18–4.00
Missing	37	1	(2.7)		
Uterus weight (g)					
<500	472	8	(1.7)	1.0	Reference
≥500	65	3	(4.6)	2.81	0.72–10.86
Missing	8	1	(12.5)		

	*N*	Postoperative complications		Odds ratio for postoperative complications	
		*n*	(%)	OR	95% CI
Total	545	46	(8.4)		
BMI (kg/m^2^)					
<30	403	36	(8.9)	1.0	Reference
≥30	105	9	(8.6)	0.96	0.44–2.05
Missing	37	1	(2.7)		
Uterus weight (g)					
<500	472	41	(8.7)	1.0	Reference
≥500	65	4	(6.2)	0.69	0.24–1.99
Missing	8	1	(12.5)		

**FIGURE 1:**
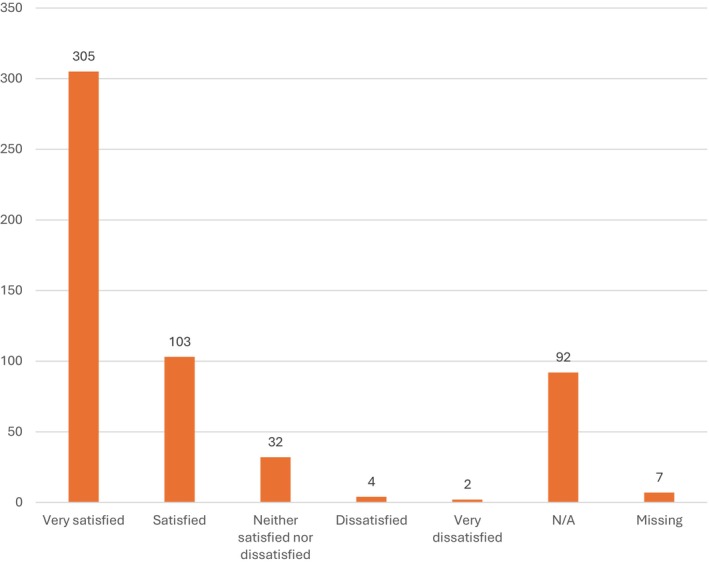
Patient satisfaction 1 year after vNOTES hysterectomy. N/A, not applicable.

In the 1‐year follow‐up questionnaire the patients were asked about their satisfaction after surgery. The results are presented in Figure 1, 92 patients answered that the question was not applicable (N/A). When these answers were removed 90.0% of the patients (*n* = 408) were very satisfied/satisfied and 1.3% of the patients (*n* = 6) were very dissatisfied/dissatisfied with the result after surgery. Among the dissatisfied patients, one had a severe postoperative complication, and one had a mild postoperative complication. The remaining four patients had no postoperative complications.

The intraoperative costs including sealing device, optics, trocars, vNOTES gelpath, gas insufflators, and sterilization costs were in 2022 approximately 490€ per patient for vNOTES hysterectomies.

## 4 DISCUSSION

This mainly descriptive study reports the surgical outcome, costs, and patient satisfaction of the 545 first vNOTES hysterectomies for benign indications in Sweden. The total intraoperative complication rate was 2.2% and the total postoperative complication rate was 8.4%. The intraoperative costs for vNOTES hysterectomies were 490€ per patient. The 1‐year follow‐up after surgery showed that 90% of the patients were satisfied or very satisfied with the result.

The total intraoperative and postoperative complication rates were similar compared to studies of other minimally invasive hysterectomy techniques, despite being in a surgical implementation phase.^1^, ^24^, ^25^ Surgical time, intraoperative blood loss and conversions were in analogy with the results of previous observational vNOTES studies.^26^, ^27^

The most common intraoperative complication was bladder injury (1.28%). Previous studies have shown a bladder injury rate of 1.2%–1.6%.^26^, ^28^, ^29^ Most of the bladder injuries were defined as mild complications. In the GynOp registry the surgeon can decide if the bladder injury is considered as minor or major with pre‐set tick box alternatives. There are no predefined circumstances to differentiate between mild and severe intraoperative complications, but to the surgeon's discretion to decide. A higher risk of bladder injury has been shown in vaginal hysterectomies compared to laparoscopic hysterectomies.^30^ The possibility of an endoscopic entrance to the vesico‐uterine pouch, rather than a classical manual anterior colpotomy when performing a vNOTES hysterectomy can probably reduce the risk of bladder injury in comparison to VH.

Three ureteral injuries were reported; all conservatively treated with ureteral stents. Stuart et al. reported a lower incidence of ureteral injury in a large international cohort.^29^ The Alexis ring used in vNOTES hysterectomies lateralizes the ureter which protects the ureter from damage. The ureteral injuries found in our study are believed to be an effect of the placement of the McCall sutures, which were used to prevent apical prolapse. The procedure involves placing sutures through the posterior vaginal cuff, peritoneum, and uterosacral ligaments to elevate and support the vaginal apex. If the McCall suture is placed too lateral, it could affect the ureter. The closure of the vaginal cuff in a vNOTES hysterectomy is identical as in a vaginal hysterectomy. Some vNOTES surgeons have therefore continued with the McCall suture when performing a vNOTES hysterectomy even though it is not part of a standard vNOTES hysterectomy.

This study represents the surgeons´ learning curve data of vNOTES hysterectomies. The results should therefore be seen in the light of vNOTES being a new technique. Between 2021 and 2023 eight hospitals have started with vNOTES successively, which can explain why one hospital has performed most of the cases while others only have performed a few. All 23 surgeons have participated in a standardized vNOTES course in Sweden or in Belgium. The surgeons were gynecologists specialized in gynecological surgery with varying skill levels, ranging from highly experienced capable of performing a hysterectomy independently to less experienced surgeons requiring supervision. None of the surgeons in Sweden had performed vNOTES hysterectomies before the implementation.

The postoperative complications were mainly urinary tract infections and other low‐grade infections. These conditions are sometimes lost to follow‐up since the patients seek medical attention in primary care. The GynOp registry has a high coverage, and the non‐responders are few, which could make this data more reliable compared to other registries. We reported 10 vaginal vault hematomas in 545 patients (1.8%), which is in line with other studies.^29^ The risk of developing vaginal vault hematomas seems to be lower compared to VH, presumably as it is possible to address bleeding endoscopically prior to closing the vaginal cuff.^31^

There were no significant differences between the number of complications among patients with BMI <30 compared to BMI ≥30 or patients with uterus weight <500 g compared to uterus weight ≥500 g. This could possibly depend on the small numbers of complications. Another possible explanation to why no differences were seen could be that difficult cases with high BMI and large uteri are usually performed by the more experienced surgeons. The mean uterus weight was 252 g and the largest uterus weighed 2935 g. More than 12% of the hysterectomies performed during the implementation period had a weight over 500 g, showing that vNOTES is a feasible minimally invasive alternative for large uteri.

In the annual report from the Swedish GynOp registry published 2024 9.2% of all benign hysterectomies were performed via vNOTES (*n* = 361).^1^ Vaginal hysterectomy was performed in 8.3% (*n* = 325), laparoscopic hysterectomy in 22.5% (*n* = 882) and abdominal hysterectomy in 23.8% (*n* = 935) of the cases. Robot assisted hysterectomy was performed in 36.2% of the cases (*n* = 1420). After the introduction of vNOTES in Sweden there has been almost a duplication in the total number of vaginally performed hysterectomies. vNOTES had the shortest time to activities of daily living (ADL) and shortest hospitalization compared to the other techniques. The mean surgical time for vNOTES was lower than for laparoscopic and robotic hysterectomies in Sweden but higher than for vaginal hysterectomies. The increased surgical time compared to VH is potentially due to the surgeons´ learning curve. Complex hysterectomies (large uteri, known adhesions) that traditionally are not performed vaginally were more commonly performed by vNOTES, which also could influence the surgical time.

Our study reports high patient satisfaction. The patients were asked about their satisfaction 1 year after surgery. Ninety‐two patients answered that the question was not applicable (N/A), presumably because they did not have any symptoms before surgery. If the indication was cervical dysplasia or endometrial pathology the patient might not have any symptoms. When these answers were removed 90.0% of the patients (*n* = 408) were very satisfied/satisfied with the result.

Cost‐effectiveness is an important matter when introducing a new surgical technique. Our study showed that the intraoperative costs for vNOTES hysterectomies were 490€ per patient. In a previous study an economic overview was made comparing AH, VH/vNOTES, TLH, and RH.^8^ The study showed that vNOTES is cost neutral compared to vaginal hysterectomies and cheaper than TLH, RH, and AH if hospitalization and surgical time is included and if a sealing device is used for vaginal hysterectomy.

A strength of this study is that it shows the surgical outcome after the implementation of vNOTES hysterectomies for a whole country with 23 surgeons from eight different hospitals. It also reports patient satisfaction 1 year after surgery. The data was prospectively collected from the GynOp registry which has high coverage. All hospitals that performed vNOTES hysterectomies in Sweden between 2021 and 2023 were included. Both large and small hospitals were participating, and the surgeons' experiences are therefore presumably variable. This possibly makes the results generalizable.

A weakness of our study is that it is a retrospective cohort study rather than a RCT, with inherent bias due to the chosen surgical method. It is prudent to choose easy cases when introducing a new technique. However, the background characteristics show that the rates of previous cesarean section/abdominal surgery, obesity, and nulliparity were higher compared to other studies when VH was performed.^8^ Recently a study protocol for a large multicenter RCT comparing vaginal hysterectomy, vNOTES hysterectomy and laparoscopic hysterectomy has been published.^32^ Patients are included from two Swedish hospitals to this study.

Another weakness of our study is that injuries can be reported both as a mild and a severe intraoperative complication in the GynOp registry. The categorization is not validated so there can be misclassifications of degree of complication. It is up to the surgeon to decide the type of intraoperative complication, which makes it more subjective. However, we believe that an intraoperative complication of importance also leads to a postoperative complication (classified according to Clavien Dindo).

## 5 CONCLUSION

This mainly descriptive study shows that the implementation of vNOTES hysterectomies in Sweden has been safe with similar complication rates compared to studies of other minimally invasive hysterectomy techniques. Surgical time, intraoperative blood loss and conversions are in analogy with the results of previous observational vNOTES studies. The 1‐year follow‐up after surgery shows that 90% of the patients were satisfied or very satisfied with the result.

## AUTHOR CONTRIBUTIONS

JW and AST ‐ design of study, applying for ethical approval, data preparation, statistical analysis, clinical interpretation, writing and amending manuscript, submitting for publication. KK ‐ data preparation, statistical analysis, amending manuscript. SE and JB ‐ design of study, clinical interpretation, amending manuscript. All authors contributed to the interpretation of the data and read and approved the final version of the article for publication.

## FUNDING INFORMATION

The study was supported by grants from Stig and Ragna Gorthon foundation. The funders had no role in the study design, data analysis, data interpretation, or writing the report.

## CONFLICT OF INTEREST STATEMENT

Johanna Wagenius, Andrea Stuart, and Jan Baekelandt declare consultancy for Applied Medical.

## ETHICS STATEMENT

The study was approved on October 11, 2023 by the Swedish Ethical Review Authority (project number 2023‐05040‐01).

## Data Availability

Research data are not shared.
